# Mulheres de Meia-Idade e Mortalidade Pós-Infarto: Um Grupo Vulnerável? Evidências de Mundo Real em uma Coorte do Sistema Único de Saúde

**DOI:** 10.36660/abc.20250296

**Published:** 2025-11-26

**Authors:** Edson Marcos Campos Lessa, Carolina Perin Maia da Silva, Tatiana Lorena da Luz Kaestner, Cecília Rubini Rocha, Gabriela Redivo Stroher, Gabriele da Silva, Giovanni Augusto de Oliveira Baccin, Marcia Olandoski, Andre Bernardi, José Rocha Faria

**Affiliations:** 1 Pontifícia Universidade Católica do Paraná Curitiba PR Brasil Pontifícia Universidade Católica do Paraná, Curitiba, PR – Brasil

**Keywords:** Pessoa de Meia-Idade, Mortalidade, Infarto

## Abstract

**Fundamento:**

As doenças cardiovasculares representam a principal causa de morte no Brasil, sendo o infarto agudo do miocárdio (IAM) responsável por parcela expressiva desses óbitos. Mulheres tendem a sofrer IAM em idade mais avançada e na presença de maior número de comorbidades, fatores que podem impactar negativamente o prognóstico. Evidências produzidas no contexto local são fundamentais para orientar estratégias específicas no âmbito do sistema público de saúde.

**Objetivo:**

Avaliar a influência da idade e do sexo na mortalidade pós-IAM em pacientes atendidos pelo Sistema Único de Saúde.

**Métodos:**

Este estudo de coorte retrospectivo foi conduzido com pacientes residentes em Curitiba e internados por IAM (código I21 da 10ª revisão da Classificação Internacional de Doenças) de 2008 a 2015. Dados sobre óbitos foram obtidos do Sistema de Informação sobre Mortalidade. Foram analisadas as mortalidades intra-hospitalar, em 6 e 12 meses e ao final do seguimento. As variáveis idade (tanto de forma contínua quanto categorizada) e sexo, bem como a interação entre ambas, foram incluídas na análise. A significância estatística foi fixada em 5% (p < 0,05).

**Resultados:**

A amostra incluiu 4.896 pacientes (idade média de 62 ± 12,4 anos; 34,1% mulheres), com seguimento médio de 50,9 meses. A mortalidade total foi de 29,5%. As mulheres sofreram IAM, em média, 5 anos mais tarde do que os homens (65,1 vs. 60,3 anos; p < 0,001) e apresentaram maior mortalidade no seguimento (p < 0,001). Contudo, na análise multivariada, apenas a idade manteve associação significativa com a mortalidade. A análise por faixa etária revelou maior risco de morte entre mulheres com 45-54,9 anos (p = 0,004).

**Conclusão:**

A idade mais avançada contribui para o pior prognóstico pós-IAM observado entre mulheres, em comparação aos homens. Entretanto, mulheres no início da meia-idade apresentaram risco aumentado de morte, configurando um possível grupo de vulnerabilidade que requer atenção específica.

## Graphical abstract

**Figura Central:**
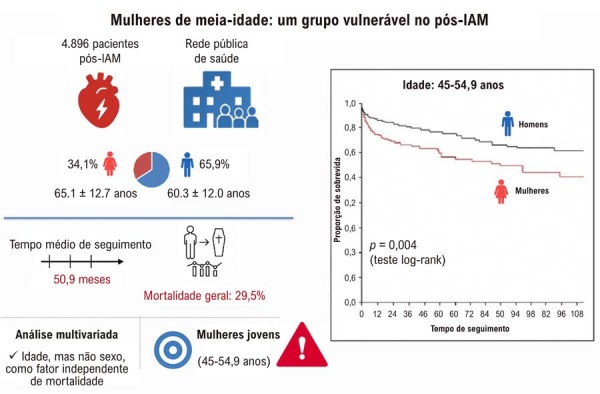
Mulheres de Meia-Idade e Mortalidade Pós-Infarto: Um Grupo Vulnerável? Evidências de Mundo Real em uma Coorte do Sistema Único de Saúde

**Figura Central:** Mulheres de Meia-Idade e Mortalidade Pós-Infarto: Um Grupo Vulnerável? Evidências de Mundo Real em uma Coorte do Sistema Único de Saúde

*IAM: infarto agudo do miocárdio.*

## Introdução

A doença cardiovascular (CV) permanece há mais de 50 anos como a principal causa de morte no Brasil, sendo os eventos coronarianos — em sua maioria, o infarto agudo do miocárdio (IAM) — responsáveis pela maior parte desses óbitos.^1^ Embora a mortalidade por eventos coronarianos apresente declínio nas regiões mais desenvolvidas, observa-se aumento dessa taxa em outras áreas.^2^ No Brasil, estima-se que ocorram anualmente 300-400 mil casos de IAM, com 1 óbito registrado a cada 5-7 ocorrências.^3^

Diversos fatores estão associados a pior prognóstico pós-IAM. Alguns fatores psicossociais, por exemplo, não apenas aumentam o risco para o evento agudo,^4^ mas também podem agravar sua evolução, como ocorre com baixa renda e menor escolaridade.^5^ Alterações como depressão também se associam a piores desfechos.^6^ Contudo, ainda há debate sobre a interação de 2 variáveis clínicas elementares no pós-IAM: idade e sexo.^7^

Pesquisas anteriores demonstram associação positiva entre idade avançada e maior mortalidade pós-IAM. Em relação ao sexo, porém, os resultados obtidos em diferentes países permanecem inconsistentes, com alguns trabalhos apontando maior mortalidade entre as mulheres.^7 - 13^ Tal achado pode estar relacionado a fatores clínicos, uma vez que em geral as mulheres sofrem IAM em idade mais avançada, bem como apresentam maior número de comorbidades e sintomas atípicos. Além disso, fatores ligados à assistência em saúde também podem contribuir: o diagnóstico nelas tende a ser mais tardio e, mesmo quando realizado em tempo oportuno, há menor frequência de indicação de terapia de reperfusão e de tratamento medicamentoso baseado em evidências. Quando se ajusta para essas covariáveis, os estudos mostram resultados conflitantes sobre o real impacto do sexo na mortalidade pós-IAM.^8 - 14^

Considerando que a evolução pós-IAM é determinada por múltiplos fatores clínicos e sociais, o eventual pior prognóstico em mulheres deve ser avaliado em contextos específicos. A elaboração de estratégias para modificar esse cenário, caso confirmada a desigualdade, depende do conhecimento da realidade local. A maior parte das evidências que apontam pior desfecho em mulheres provém de estudos controlados ou de coortes internacionais. No Brasil, especialmente no sistema público de saúde, há escassez de dados de mundo real.

Diante dessa realidade, o presente estudo teve como objetivo avaliar a influência da idade e do sexo na mortalidade de pacientes admitidos com diagnóstico de IAM em hospitais da rede pública de Curitiba.

## Métodos

Este estudo de coorte retrospectiva foi conduzido a partir da base de dados da Secretaria Municipal de Saúde (SMS) da cidade de Curitiba, estado do Paraná, Brasil. A amostra foi composta por indivíduos internados em hospitais da rede pública de Curitiba, entre janeiro de 2008 e dezembro de 2015, com diagnóstico principal de IAM — diagnóstico codificado como I21 de acordo com a 10ª revisão da Classificação Internacional de Doenças —, residentes em Curitiba e com idade igual ou superior a 18 anos. Foram excluídos pacientes com dados duplicados ou inconsistentes entre as datas de internação e óbito, bem como aqueles residentes em outros municípios.

As variáveis de interesse foram idade e sexo. A idade foi analisada tanto como variável contínua quanto categorizada em faixas etárias definidas arbitrariamente: < 45 anos; 45-54,9 anos; 55-64,9 anos; 65-79,9 anos; e ≥ 80 anos. O sexo foi definido como variável biológica,^15^ registrada na guia de internação (informação habitualmente derivada do documento de identificação).

Os dados referentes à internação por IAM e às características demográficas dos pacientes foram obtidos por meio dos registros de autorização de internação hospitalar, que incluem datas de admissão e alta, desfecho, hospital de internação e código correspondente. A análise da mortalidade foi realizada a partir do banco de dados do Sistema de Informação sobre Mortalidade (SIM), de abrangência nacional e centralizado no Ministério da Saúde, sendo considerado óbito quando havia registro no SIM ou na base da SMS. Foram identificados óbitos ocorridos até dezembro de 2015. Os desfechos avaliados foram mortalidade intra-hospitalar, em 6 e 12 meses, e ao final do seguimento.

O primeiro episódio de internação foi considerado como internação basal. A data de início do seguimento (T_0_) correspondeu à data dessa primeira internação. Para casos censurados (pacientes vivos), o término do seguimento foi fixado em 30 de junho de 2016, data de atualização do banco do SIM utilizado no presente estudo.

O projeto foi aprovado pelo Comitê de Ética em Pesquisa da Pontifícia Universidade Católica do Paraná (protocolo nº 64/2016) e pelo Comitê de Ética da SMS (parecer nº 1.647.450).

### Análise estatística

As variáveis contínuas foram descritas por médias e desvios-padrão ou por medianas e amplitudes. As variáveis categóricas foram apresentadas como frequências absolutas e percentuais. A sobrevida foi estimada por meio de curvas de Kaplan-Meier e comparada utilizando o teste de log-rank. Para a análise multivariada, foi ajustado um modelo de regressão de Cox, seguido do teste de Wald. A medida de associação estimada foi a *hazard ratio* (HR), com respectivos intervalos de confiança de 95% (IC 95%). Valores de p < 0,05 foram considerados estatisticamente significativos. As análises foram realizadas no *software* Stata, versão 14.1.

## Resultados

Foram inicialmente identificados 7.066 pacientes internados por IAM entre janeiro de 2008 e dezembro de 2015. Foram excluídos 151 casos devido a dados duplicados ou inconsistentes de internação, 4 casos por idade inferior a 18 anos e 2.015 casos por não residirem em Curitiba — condição que impediria o registro de óbitos no SIM.

**Figura 1:**
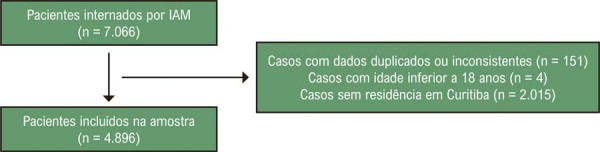
– Fluxograma do processo de seleção da amostra. IAM: infarto agudo do miocárdio.

Após as exclusões, permaneceram elegíveis para o estudo 4.896 pacientes ( Figura 1 ).

**Figura 2:**
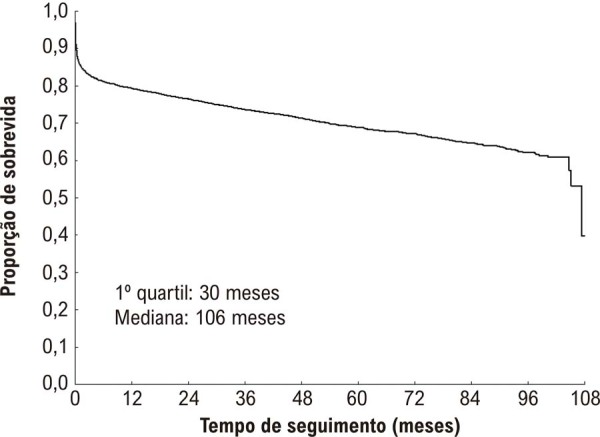
– Curva de sobrevida global da amostra.

A amostra foi composta por 34,1% de mulheres, e a idade média dos pacientes no momento do IAM foi de 62 ± 12,4 anos. O tempo médio de seguimento foi de 50,9 meses, com mortalidade geral de 29,5% nesse período — a maior taxa de óbitos registrada no primeiro ano após o evento ( Figura 2 ). Houve redução expressiva no número de pacientes em acompanhamento nos períodos finais do seguimento.

**Figura 3:**
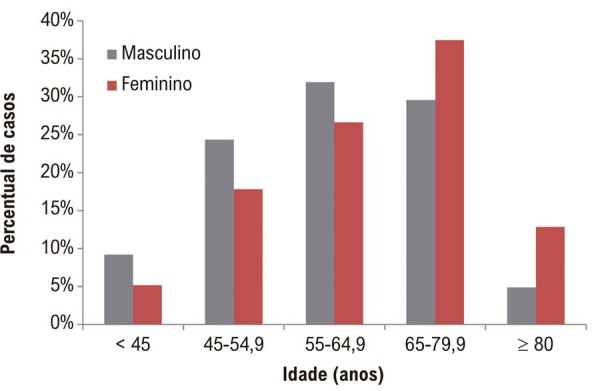
– Distribuição dos casos de infarto agudo do miocárdio por faixa etária e sexo.

Cerca de 15% dos casos de IAM ocorreram em indivíduos jovens (< 45 anos; 7,8%) ou muito idosos (≥ 80 anos; 7,6%). A idade média das mulheres no momento do IAM foi aproximadamente 5 anos superior à dos homens (65,1 ± 12,7 anos vs. 60,3 ± 12,0 anos; p < 0,001). Além disso, a análise da distribuição por faixa etária revelou que uma proporção maior de mulheres apresentou IAM nas faixas etárias mais elevadas ( Figura 3 ).

**Figura 4:**
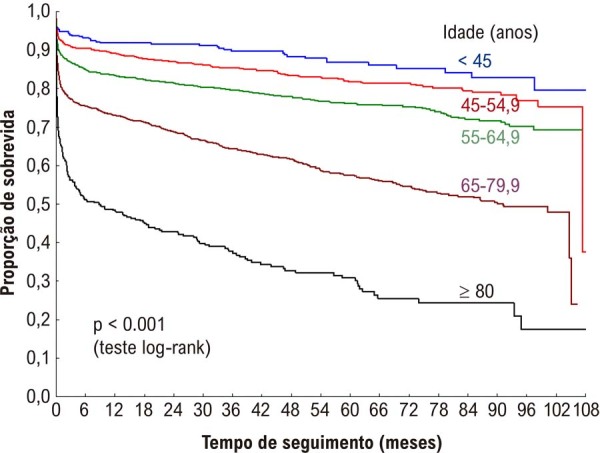
– Sobrevida após a admissão por infarto agudo do miocárdio, de acordo com a faixa etária.

A idade mostrou-se um determinante de pior prognóstico (p < 0,001), com redução progressiva da sobrevida em pacientes das faixas etárias mais elevadas ( Figura 4 ).

**Figura 5:**
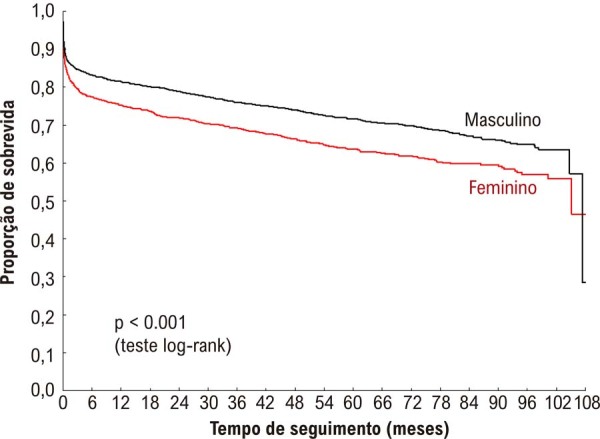
– Estratificação prognóstica de acordo com o sexo.

**Figura 6:**
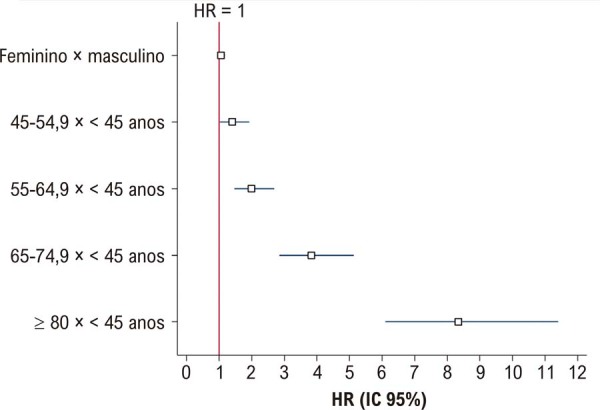
– Análise multivariada do efeito da idade e do sexo na sobrevida global. HR: hazard ratio.

A Figura 5 apresenta as curvas de sobrevida segundo o sexo, evidenciando pior prognóstico para as mulheres após o evento agudo (p < 0,001). No entanto, a análise multivariada, ajustada para idade e sexo, não identificou diferença estatisticamente significativa na taxa de mortalidade entre homens e mulheres. Em relação à idade, observou-se aumento progressivo no risco de morte nas faixas etárias mais elevadas ( Figura 6 ).

### Análise da evolução pós-IAM por sexo e faixa etária

**Figura 7:**
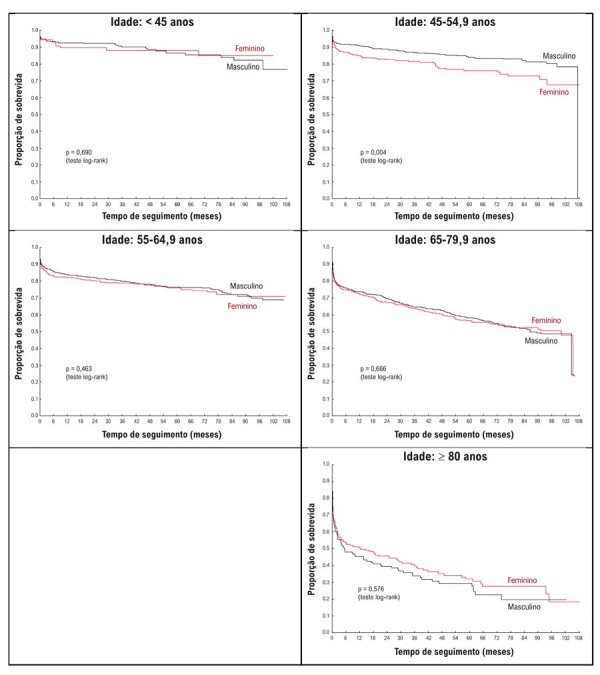
– Estratificação prognóstica segundo o sexo e a faixa etária.

A análise das curvas de sobrevida de homens e mulheres, estratificados por faixa etária, revelou evolução semelhante na maioria dos grupos etários, com exceção da faixa 45-54,9 anos. Nesse grupo, as mulheres apresentaram mortalidade significativamente maior em comparação aos homens (p = 0,004) ( Figura 7 ).

## Discussão

Até onde sabemos, este estudo fornece a primeira visão de mundo real sobre a evolução clínica de pacientes tratados por IAM em Curitiba, um importante centro urbano brasileiro. Por se tratarem de dados provenientes da rede pública de saúde — responsável pelo atendimento exclusivo de 71,5% da população brasileira^16^ —, sua interpretação pode contribuir para o desenvolvimento de estratégias voltadas à redução do impacto dos eventos coronarianos no país.

Embora a maior mortalidade pós-IAM observada entre as mulheres possa ser atribuída, em parte, ao fato de estas apresentarem o evento, em média, quase 5 anos mais tarde que os homens, a diferença significativa identificada no grupo de pacientes nos anos iniciais da meia-idade (45-54,9 anos) pode indicar a existência de um subgrupo feminino mais vulnerável ( Figura Central ).

Em nossa coorte, a idade associou-se de forma significativa à mortalidade. Esse achado está em concordância com estudos prévios, que também demonstram forte correlação entre idade avançada e incidência de novos eventos.^10 , 11 , 17^ Como mencionado, a idade parece explicar a diferença de mortalidade observada entre homens e mulheres, uma vez que a análise multivariada indicou que apenas a idade, e não o sexo, manteve-se como fator independente associado a maior mortalidade.

No momento do IAM, as mulheres tendem a ser mais idosas e, consequentemente, apresentam maior número de comorbidades. Além disso, parecem ser menos propensas a receber terapias medicamentosas baseadas em evidências ou tratamento de reperfusão.^9 , 12 - 14 , 17 - 22^ Entretanto, alguns estudos descrevem o sexo feminino como fator de risco independente para mortalidade em idades inferiores a 65 anos, faixa etária na qual se reporta mortalidade 11%-30% maior em comparação aos homens.^8 , 12 , 13 , 19 - 23^ Nosso achado de maior mortalidade entre mulheres com 45-54,9 anos está em consonância com essa hipótese.

Esse fenômeno, descrito como “paradoxo de gênero” em mulheres jovens, tem sido objeto de diversas investigações. Tal grupo tende a apresentar menos fatores de risco CV,^21^ menor incidência de doença arterial coronariana (DAC) multiarterial,^17 , 21^ melhor resposta à reperfusão após angioplastia^21^ e maior incidência de DAC não obstrutiva^15 , 17 , 21^ — todos fatores que, em tese, estariam associados a melhor prognóstico na população geral.

Recentemente, características hormonais próprias do sexo feminino — como uso de contraceptivos, gravidez e menopausa —, além de fatores como sintomas atípicos, subdiagnóstico, subtratamento e ausência de acompanhamento adequado, têm sido apontados como relevantes na determinação do risco CV em mulheres.^24^ A menopausa, período de transição fisiológica, tem sido correlacionada a maior risco CV, embora os mecanismos envolvidos ainda não estejam totalmente elucidados.^25^

A menor incidência de reperfusão, já amplamente documentada, costuma ser atribuída ao maior risco de sangramento em mulheres, presente em todas as faixas etárias e até 2 vezes mais elevado em comparação aos homens. Nas pacientes jovens, esse cenário é agravado pela maior incidência de DAC não obstrutiva.^19^

Fatores vasculares também têm sido apontados como possíveis explicações para o pior prognóstico. Independentemente da faixa etária, as mulheres tendem a apresentar menor extensão de DAC, vasos de menor calibre, menor circulação colateral e, consequentemente, menor pré-condicionamento isquêmico.^8 , 17^ Associado a isso, o maior tempo para procurar atendimento hospitalar e para a realização do diagnóstico aumenta a vulnerabilidade à isquemia aguda.^17^

Outros autores sugerem que aspectos como maior incidência de depressão, estresse psicológico, declínio funcional e redução da qualidade de vida possam influenciar a mortalidade, especialmente entre mulheres com menos de 65 anos.^8 , 14^

Além disso, nas mulheres jovens, o mecanismo de apresentação da placa aterosclerótica costuma diferir daquele observado nos homens. Nelas, é mais frequente a erosão da placa com embolização microvascular, o que pode resultar em disfunção e reatividade coronariana anormal. Já nos homens jovens, o padrão mais comum é a ruptura da placa com trombose aguda — evento associado a maior risco de óbito pré-hospitalar e que pode atuar como variável de confusão na análise da maior mortalidade feminina.^17 , 19 , 21^

### Limitações do estudo

Por ser um estudo de coorte retrospectivo baseado na análise de uma base de dados pública, este trabalho pesquisa apresenta limitações inerentes à qualidade do preenchimento das informações. A ausência de dados sobre comorbidades, tipos de tratamento instituídos e achados angiográficos impossibilitou correlacionar a maior mortalidade observada entre mulheres com 45-54,9 anos com os fatores já identificados em pesquisas anteriores.

## Conclusão

Na população atendida pelos serviços públicos de saúde de Curitiba, as mulheres apresentaram pior prognóstico no período pós-IAM. Essa maior mortalidade parece estar relacionada ao fato de sofrerem o evento, em média, quase 5 anos mais tarde que os homens. Entretanto, em uma faixa etária mais jovem, a mortalidade feminina foi significativamente superior. Esses achados sugerem que mulheres nos anos iniciais da meia-idade podem representar um grupo particularmente vulnerável, sendo necessários novos estudos para investigar as razões subjacentes. A redução dessas disparidades de sexo requer educação médica continuada, melhor compreensão das barreiras ao tratamento adequado e intervenções direcionadas aos determinantes sociais da saúde.^26^
