# Supplementary material for: Mulheres de Meia-Idade e Mortalidade Pós-Infarto: Um Grupo Vulnerável? Evidências de Mundo Real em uma Coorte do Sistema Único de Saúde
Source: Arq Bras Cardiol. 2025 Nov 26;122(10):e20250296. [Article in Portuguese] doi: 10.36660/abc.20250296 (PMC13395323; doi:10.36660/abc.20250296)
Supplement: Translation

# Translation: Middle-Aged Women and Post-Myocardial Infarction Mortality: A Vulnerable Group? Real-World Evidence from a Brazilian Public Health System Cohort

**DOI:** 10.36660/abc.20250296i

**Authors:** Edson Marcos Campos Lessa, Carolina Perin Maia da Silva, Tatiana Lorena da Luz Kaestner, Cecília Rubini Rocha, Gabriela Redivo Stroher, Gabriele da Silva, Giovanni Augusto de Oliveira Baccin, Marcia Olandoski, Andre Bernardi, José Rocha Faria

**Affiliations:** 1 Brazil Pontifícia Universidade Católica do Paraná, Curitiba, PR – Brazil

**Keywords:** Middle Aged, Mortality, Infarction

## Abstract

**Background:**

Cardiovascular diseases are the leading cause of death in Brazil, with acute myocardial infarction (MI) accounting for a significant share of these fatalities. Women tend to experience MI at older ages and in the presence of more comorbidities — factors that may negatively impact prognosis. Locally generated evidence is essential to guide targeted strategies within the public health care system.

**Objective:**

To assess the influence of age and sex on post-MI mortality among patients treated within the Brazilian Unified Health System.

**Methods:**

This retrospective cohort study included patients residing in Curitiba who were hospitalized for MI (International Classification of Diseases, 10th Revision code I21) between 2008 and 2015. Mortality data were obtained from the Brazilian Mortality Information System. In-hospital mortality, as well as mortality at 6 and 12 months and at the end of follow-up, were analyzed. Age (both as a continuous and categorical variable) and sex, as well as their interaction, were included in the analysis. Statistical significance was set at 5% (p < 0.05).

**Results:**

The sample included 4,896 patients (mean age 62 ± 12.4 years; 34.1% women), with an average follow-up of 50.9 months. Overall mortality was 29.5%. On average, women experienced MI 5 years later than men (65.1 vs. 60.3 years; p < 0.001) and had higher follow-up mortality (p < 0.001). However, in the multivariate analysis, only age remained significantly associated with mortality. Age-stratified analysis revealed a higher risk of death among women aged 45-54.9 years (p = 0.004).

**Conclusion:**

Older age contributes to the worse post-MI prognosis observed among women compared to men. However, women in early middle age showed an increased risk of death, representing a potentially vulnerable group requiring specific attention.

## Introduction

For over 50 years, cardiovascular (CV) diseases have been the leading cause of death in Brazil, with most of these fatalities being due to coronary events — primarily acute myocardial infarction (MI).^1^Although coronary mortality rates have declined in more developed regions, they have increased in other areas.^2^In Brazil, it is estimated that there are 300,000-400,000 MIs each year, with 1 death recorded for every 5-7 events.^3^

Several factors are associated with a worse prognosis after MI. For instance, psychosocial factors not only increase the risk of an acute event^4^but they may also worsen its course. This is seen with low income and lower educational attainment.^5^Depression and other conditions are also linked to poorer outcomes.^6^However, the interaction between 2 key clinical variables — age and sex — in the post-MI setting is still debated.^7^

Previous research has demonstrated a positive association between older age and higher post-MI mortality. Regarding sex, however, findings across countries remain inconsistent. Some studies have reported higher mortality among women.^7 - 13^This finding may be related to clinical factors. Women generally experience MI at older ages, with a higher burden of comorbidities, and with atypical symptoms. Additionally, health care-related factors may play a role: diagnosis in women tends to occur later. Even when timely, reperfusion therapy and evidence-based pharmacological treatment are less frequently indicated. Studies show conflicting results on the actual impact of sex on post-MI mortality when adjusting for these covariates.^8 - 14^

Because post-MI outcomes are influenced by multiple clinical and social factors, the potentially worse prognosis in women must be evaluated in specific contexts. Should this disparity be confirmed, developing strategies to address it requires an understanding of the local reality. Most evidence indicating poorer outcomes for women originates from controlled trials or international cohorts. In Brazil, particularly within the public health care system, real-world data remains scarce.

Therefore, the present study aimed to assess the influence of age and sex on mortality among patients admitted with a diagnosis of MI to public hospitals in the city of Curitiba.

## Methods

This retrospective cohort study was conducted using data from the Municipal Health Secretariat (SMS) of the city of Curitiba, state of Paraná, Brazil. The sample comprised individuals admitted to public hospitals in Curitiba between January 2008 and December 2015 with a primary diagnosis of MI — coded as I21 according to the International Classification of Diseases, 10th Revision — who resided in Curitiba and were aged 18 years or older. Patients with duplicate records, inconsistent dates of admission and death, or residence in other municipalities were excluded.

The variables of interest were age and sex. Age was analyzed both as a continuous variable and as a categorical variable, divided into arbitrarily defined age groups: < 45 years; 45-54.9 years; 55-64.9 years; 65-79.9 years; and ≥ 80 years. Sex was defined as a biological variable,^15^recorded in the hospital admission form (usually derived from the patient’s identification document).

Data on MI admissions and patient demographic characteristics were obtained from hospital admission authorization records, which include admission and discharge dates, outcomes, admitting hospital, and corresponding diagnostic codes. Mortality analysis was based on the Mortality Information System (SIM) database, which has national coverage and is centralized by the Brazilian Ministry of Health. Death was considered when recorded in either the SIM or the SMS database. Deaths up to December 2015 were included. The evaluated outcomes were in-hospital mortality, 6-month and 12-month mortality, and mortality at the end of follow-up.

The first admission was considered the baseline hospitalization. The start of follow-up (T_0_) corresponded to the date of this first admission. For censored cases (patients alive), follow-up ended on June 30, 2016, which was the date of the SIM database update used in this study.

The study was approved by the Research Ethics Committee at Pontifícia Universidade Católica do Paraná (protocol No. 64/2016) and by the Ethics Committee at SMS (approval No. 1,647,450).

### Statistical analysis

Continuous variables were described as means and standard deviations or as medians and ranges. Categorical variables were presented as absolute frequencies and percentages. Survival was estimated using Kaplan-Meier curves and compared with the log-rank test. For multivariate analysis, a Cox regression model was fitted, followed by the Wald test. The measure of association was the hazard ratio (HR) with corresponding 95% CIs. P values < 0.05 were considered statistically significant. Analyses were performed using Stata software, version 14.1.

## Results

A total of 7,066 patients hospitalized for MI between January 2008 and December 2015 were initially identified. Of these, 151 cases were excluded due to duplicate or inconsistent admission records, 4 cases were excluded due to age under 18 years, and 2,015 cases were excluded because the patients were not residents of Curitiba — a condition that would prevent death registration in the SIM.

After these exclusions, 4,896 patients remained eligible for the study ( Figure 1 ).

**Figure 1:** – Flowchart of the sample selection process. MI: myocardial infarction.

The sample consisted of 34.1% women, and the mean age of patients at the time of MI was 62 ± 12.4 years. The mean follow-up duration was 50.9 months, with an overall mortality rate of 29.5% during this period — the highest mortality occurring within the first year after the event ( Figure 2 ). A marked reduction in the number of patients under follow-up was observed in the later stages of the follow-up period.

**Figure 2:** – Overall survival curve of the sample.

Approximately 15% of MI cases occurred in either young individuals (< 45 years; 7.8%) or very older individuals (≥ 80 years; 7.6%). The mean age of women at the time of MI was about 5 years higher than that of men (65.1 ± 12.7 years vs. 60.3 ± 12.0 years; p < 0.001). Moreover, the age group distribution analysis showed a higher proportion of women experiencing MI in the older age brackets ( Figure 3 ).

**Figure 3:** – Distribution of acute myocardial infarction cases by age group and sex.

Age was a determinant of poorer prognosis (p < 0.001), with progressively lower survival observed in patients from older age groups ( Figure 4 ).

**Figure 4:** – Survival after admission for acute myocardial infarction, according to age group *.*

Figure 5 shows the survival curves by sex, revealing a poorer prognosis for women after the acute event (p < 0.001). However, multivariate analysis adjusted for age and sex did not identify a statistically significant difference in mortality rates between men and women. Regarding age, a progressive increase in the risk of death was observed in the older age groups ( Figure 6 ).

**Figure 5:** – Prognostic stratification by sex.

**Figure 6:** – Multivariate analysis of the effect of age and sex on overall survival. HR: hazard ratio.

### Post-MI outcomes by sex and age group

Analysis of the survival curves for men and women, stratified by age group, revealed similar outcomes across most age categories, except for the 45-54.9-year group. In this group, women had significantly higher mortality compared to men (p = 0.004) ( Figure 7 ).

**Figure 7:** – Prognostic stratification by sex and age group.

## Discussion

To the best of our knowledge, this study provides the first real-world overview of the clinical course of patients treated for MI in Curitiba, a major Brazilian urban center. As data originate from the public health care system — which is solely responsible for serving 71.5% of the Brazilian population^16^— its interpretation may contribute to the development of strategies aimed at reducing the burden of coronary events in Brazil.

Although the higher post-MI mortality observed among women may be partly attributed to the fact that, on average, they experience the event nearly 5 years later than men, the significant difference identified in the early middle-aged group (45-54.9 years) may indicate the presence of a more vulnerable female subgroup ( Central Illustration ).

In our cohort, age was significantly associated with mortality. This finding is consistent with previous studies, which also demonstrate a strong correlation between older age and the incidence of new events.^10 , 11 , 17^As mentioned, age appears to explain the mortality difference observed between men and women, since multivariate analysis indicated that only age — rather than sex — remained an independent factor associated with increased mortality.

At the time of MI, women tend to be older and, consequently, present with a higher number of comorbidities. In addition, they appear to be less likely to receive evidence-based pharmacological therapies or reperfusion treatment.^9 , 12 - 14 , 17 - 22^Some studies have described female sex as an independent risk factor for mortality among individuals under 65 years of age, a group in which mortality is reported to be 11%-30% higher compared to men.^8 , 12 , 13 , 19 - 23^Our finding of higher mortality among women aged 45-54.9 years is consistent with this hypothesis.

This phenomenon, referred to as the “gender paradox” in younger women, has been the subject of several investigations. This group tends to have fewer CV risk factors,^21^a lower incidence of multivessel coronary artery disease (CAD),^17 , 21^better response to reperfusion after angioplasty,^21^and a higher incidence of nonobstructive CAD^15 , 17 , 21^— all of which would, in theory, be associated with a more favorable prognosis in the general population.

Recently, female-specific hormonal characteristics — such as the use of contraceptives, pregnancy, and menopause — as well as factors such as atypical symptoms, underdiagnosis, undertreatment, and lack of appropriate follow-up have been identified as relevant in determining CV risk in women.^24^Menopause, a physiological transition, has been correlated with increased CV risk, although the mechanisms involved are not yet fully understood.^25^

The lower rate of reperfusion, which is well-documented, is often attributed to a higher risk of bleeding in women, a risk that is present across all age groups and is up to twice as high compared to men. Among younger women, this scenario is worsened by the higher incidence of nonobstructive CAD.^19^

Vascular factors have also been proposed as potential explanations for the worse prognosis. Regardless of age, women tend to have less extensive CAD, smaller-caliber vessels, reduced collateral circulation, and, consequently, lower ischemic preconditioning.^8 , 17^In addition, longer delays in seeking medical care and in achieving diagnosis increase vulnerability to acute ischemia.^17^

Other authors have suggested that factors such as a higher incidence of depression, psychological stress, functional decline, and reduced quality of life may influence mortality, especially among women under the age of 65.^8 , 14^

Furthermore, in younger women, the mechanism of atherosclerotic plaque presentation tends to differ from that observed in men. In women, plaque erosion with microvascular embolization is more frequent and may result in coronary dysfunction and abnormal reactivity. In contrast, the most common pattern in young men is plaque rupture with acute thrombosis — an event associated with a higher risk of pre-hospital death, which may act as a confounder in the analysis of increased female mortality.^17 , 19 , 21^

### Limitations of the study

As a retrospective cohort study based on the analysis of a public database, this study presents limitations inherent to the quality of data entry. The lack of information regarding comorbidities, types of treatment administered, and angiographic findings made it impossible to correlate the higher mortality observed among women aged 45-54.9 years with the factors previously identified in other studies.

## Conclusion

Among the population treated in public health care services in Curitiba, women had a worse post-MI prognosis. This higher mortality appears to be related to the fact that they experience the event, on average, nearly 5 years later than men. However, in a younger age group, female mortality was significantly higher. These findings suggest that women in the early years of middle age may represent a particularly vulnerable group, warranting further investigation into the underlying causes. Reducing these sex-based disparities requires continued medical education, a better understanding of the barriers to adequate treatment, and targeted interventions addressing the social determinants of health.^26^
